# Preterm birth and the timing of puberty: a systematic review

**DOI:** 10.1186/s12887-017-0976-8

**Published:** 2018-01-08

**Authors:** Evlyn James, Claire L. Wood, Harish Nair, Thomas C. Williams

**Affiliations:** 1 Royal Oldham Hospital, Rochdale Road, Oldham, UK; 2 Newcastle Hospitals NHS Foundation Trust, Newcastle upon Tyne, UK; 3 Usher Institute of Population Health Sciences and Informatics, University of Edinburgh, Edinburgh, UK; 4 Institute of Genetics and Molecular Medicine, University of Edinburgh, Western General Hospital, Crewe Road, Edinburgh, EH4 2XU UK

**Keywords:** Menarche, Follow up studies

## Abstract

**Background:**

An estimated 11% of births occur preterm, and survival is improving. Early studies suggested an association between preterm birth and earlier puberty. Given the adverse outcomes associated with early puberty this could have significant public health implications.

The objective of this review was to assess the timing of puberty after preterm birth.

**Methods:**

Pubmed, Embase, Popline, Global Health and Global Health Library were searched using terms relating to “premature birth”, “menarche”, “puberty” and “follow up studies”. Inclusion criteria were a population consisting of pubertal or post-pubertal adolescents and adults; studies which defined preterm delivery in participants and compared outcomes to those after term delivery; and a quantitative assessment of pubertal onset. Assessment of risk of bias was conducted using principles from the Critical Appraisal Study Process.

**Results:**

Our search identified 1051 studies, of which 16 met the inclusion criteria. In females, 8 studies found no association between preterm birth and the timing of menarche. Five studies found earlier onset in preterm infants, 1 found later onset, and 1 showed both earlier and later menarche, depending on birth weight. The range of effect of studies showing earlier menarche was - 0.94 to −0.07 years in the preterm group, with a median of - 0.3 years. In males, 2 studies showed earlier onset of puberty in the preterm group, 5 showed no difference, and 1 showed later onset. Most studies did not present outcomes in the form of a mean with standard deviation, precluding a meta-analysis. There was insufficient data to address potential confounding factors.

**Conclusions:**

The published evidence does not suggest that being born preterm leads to a significant acceleration in the onset of puberty. This should prove reassuring for public health purposes, and for clinicians counseling parents of infants born preterm.

**Electronic supplementary material:**

The online version of this article (10.1186/s12887-017-0976-8) contains supplementary material, which is available to authorized users.

## Background

Preterm birth is common, with an estimated 11% of infants worldwide being born at a gestational age of less than 37 weeks [1]. Survival of preterm infants born even at very early gestations is improving, [2] and thus these patients are now consistently surviving into adolescence and adulthood. It is increasingly recognized that preterm birth is an independent risk factor for adverse cardiometabolic [3] and neurodevelopmental outcomes, [4] even following birth at moderate (32–33 weeks) and late (34–36 weeks) preterm gestation. Although the precise mechanism for preterm deliveries cannot be established in most cases, [5] epidemiological studies have shown a correlation between low socio-economic status, adverse life circumstances, and an increased risk of preterm delivery [5–7]. Earlier puberty, particular in females, has also been linked with lower socioeconomic status and adverse early life circumstances [8, 9]. Like preterm birth, earlier puberty also seems to be associated with an increased risk of cardiovascular [10, 11] and metabolic [12, 13] disease in adult life. In addition, in females earlier sexual development may be linked to an increased risk of cancer, [14, 15] depression, [16] and other psychopathology later in life [14, 17].

Some authors have postulated both preterm birth and earlier puberty as part of a complex of adaptive phenotypic changes (a ‘predictive adaptive response’) made in response to a threatening developmental environment [18]. This hypothesis is supported by early data which showed that preterm birth was associated with earlier (6 months) onset of menarche, as compared to term controls [19]. To examine the hypothesis that preterm birth is associated with a stereotyped phenotypic developmental trajectory, we carried out a systematic review looking at the association between preterm birth and the timing of puberty. Given the morbidity associated with both entities, if there proved to be relationship between the two this would have significant public health consequences. In addition, this information would be important for clinicians counseling parents and eventually patients on the longer term consequences of preterm birth. We therefore asked the research question: in adolescents (Population), what are the effects of being born prematurely at <37 weeks (Exposure) versus being born at term (Comparison) on the timing of onset of puberty (Outcome), as reported in cohort, cross sectional or case control studies (Study design).

## Methods

### Searches

We carried out a systematic literature review in September 2015 using the following databases: Medline, [20] Embase, [21] and Global Health [22] (all using the OVID interface), [23] Popline [24] and Global Health Library [25]. Search strategies were generated using MESH and Emtree terms relating to “premature birth”, “menarche”, “puberty” and “follow up studies,” with input from a medical librarian. A complete list of search terms, formatted for each database, is available within the study protocol in Additional file 1: Appendix S1. The review is registered on PROSPERO, [26] CRD42015024806.

Databases were searched from 1946 onwards. Only papers with abstracts published in the Latin alphabet were reviewed, and these were translated if necessary by one of the authors (TW). We conducted reference searches of the studies which met the inclusion criteria, and contacted experts in the field in to identify further relevant studies. Two reviewers (EJ and CW) independently assessed the papers identified in the screening search using the inclusion and exclusion criteria.

### Inclusion/exclusion criteria

Studies were included if 1) the population consisted of pubertal and post-pubertal adolescents and adults, 2) they were cohort, cross sectional or case control studies, 3) they defined preterm delivery in participants and compared this to term delivery, and 4) they carried out a quantitative assessment (either by participants or study researchers) of commencement of puberty, in the form of changes on growth charts, clinical examination of Tanner stages, or age at onset of menarche (first menstrual period) .

Studies were excluded if 1) they were case reports or opinion pieces, 2) they reported on birthweight but not gestation for the patient population, or 3) they were qualitative studies that did not provide quantitative data on the age of onset of puberty.

### Data extraction, assessment of study quality and risk of bias

The following data were extracted from the studies meeting the inclusion criteria: authors, study publication date, country where the study was conducted, sex of participants, study design, study setting, definition of prematurity, number of study subjects (term and preterm), mean/median age of onset of puberty (from growth charts), Tanner stages, menarche, or age at voice breaking, and whether a statistical summary measure was calculated for the results. Data were entered onto Microsoft Excel (Microsoft Corporation, Redmond, WA, USA).

In order to assess the risk of bias within each individual study, we applied principles from the Critical Appraisal Study Process (CASP), [27] examining whether an appropriate study design had been chosen, whether the exposure and outcome were accurately measured, whether potential confounding factors were identified, and if so whether they had been adjusted for, and whether a statistical summary measure was given with the results. In order to assess the risk of publication bias, we noted whether or not each study had been published in a peer reviewed journal, and we contacted experts in the field to ascertain if there were relevant large datasets that remained unpublished.

## Results

### Searches

**Fig. 1:**
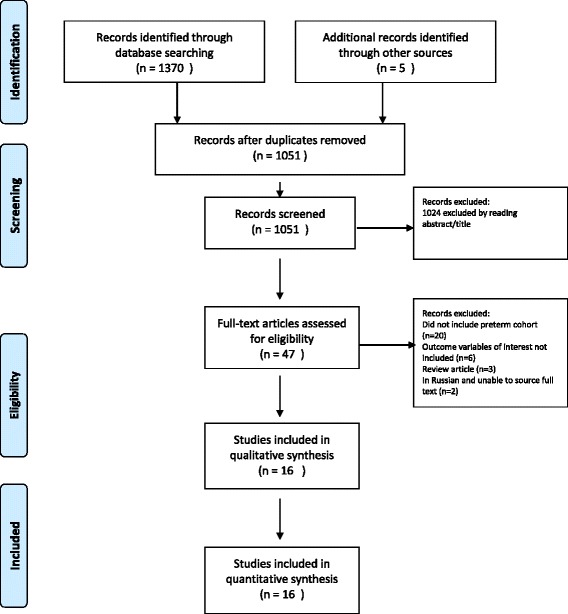
PRISMA Flow Chart

Our database search yielded 1370 records, and consultation with experts in the field identified 1 further study. Reference searches of 11 studies that met the inclusion criteria yielded 4 additional records, and after excluding duplicates a total of 1051 studies were screened. 47 of these studies were selected for full text review, of which 16 studies met the inclusion criteria. Figure 1 shows the PRISMA flowchart for the search. Four studies were from the United States; [28–31] 2 studies from Canada, [32, 33] Australia [34, 35], Finland [36, 37] and India; [19, 38] and 1 study from Hong Kong, [39] France, [40] Sweden, [41] and Turkey [42]. Participants in the studies were born between 1929 and 2003.

### Assessment of risk of bias at study and outcome level

The assessment of risk of bias is shown in Table 1. All the studies asked a clearly focused study question. Twelve of the included studies were cohort studies [19, 28, 30, 32–40] (of which 2 were nested cohorts), [32, 34] 3 were cross sectional, [29, 31, 42] and 1 was a case control; [41] in all cases the study design was appropriate, although there was variation in the identification of potential confounding factors and adjustment for these. There was variability in how the exposure (gestational age at delivery) was assessed, with 5 studies not documenting how this was calculated, [19, 35, 36, 38, 41] 4 studies relying on an assessment of gestational age from the participants or their parents, [28, 32, 40, 42] and 7 using a combination of the date of last menstrual period with ultrasound measurements if available. [29–31, 33, 34, 37, 39] There was also variability in how the outcome (age at onset of puberty or menarche) was assessed, with 5 studies using self-reporting from participants or their parents, [28, 32, 35, 40, 42] 10 studies using data from outpatients clinics, [19, 29, 31, 33–39, 41] and 1 study not documenting this process [30]. Fourteen out of the 16 studies [19, 28–31, 33–40, 42] documented possible confounding factors, although only 8 of these corrected for them in subsequent analyses [19, 28, 29, 31, 33, 36, 38, 39]. Seven studies [19, 31, 32, 34, 35, 38, 42] did not provide a statistical summary measure for the comparison between term and preterm infants, thus limiting our ability to interpret and compare results.

**Table 1:** Assessment of risk of bias

Authors	Peer reviewed?	Clearly focused study question?	Study design	Cohort recruitment acceptable? (Cohort, cross sectional studies)	Case control appropriate study method? (Case control studies)	Exposure (gestational age at birth) accurately measured?	Outcome (age at menarche/onset puberty) accurately measured?	Confounding factors identified?	Confounding factors accounted for?	Statistical summary measure given?
Atay et al.	Yes	Yes	Cross-sectional	Yes	N/A	Reported by parents	Self reported by participants or parents	Yes	No	No
Bhargava et al	Yes	Yes	Cohort	Yes	N/A	Not documented	Data from outpatient clinics	Yes	Yes	No
Chaudhari et al	Yes	Yes	Cohort	Yes	N/A	Not documented	Data from outpatient clinics	Yes.	Yes	No
D’Aloisio et al.	Yes	Yes	Cohort	Yes.	N/A	Self reported by participants or parents	Self reported by participants	Yes	Yes	Yes
Dossus et al	Yes	Yes	Cohort	Yes	N/A	Self reported by participants	Self reported by participants	Yes	No	Yes
Epplein et al	Yes	Yes	Cross-sectional	Yes	N/A	Calculated from date of LMP	Data from outpatient clinics	Yes	Yes	Yes
Ford et al	Yes	Yes	Nested cohort	Yes	N/A	Calculated from date of LMP and ultrasound measurements	Data from outpatient clinics	Yes	No	No
Hack et al.	Yes	Yes	Cohort	Control group recruited at 8 years of age	N/A	Calculated from date of LMP	Not documented	Yes	No	Yes
Hui et al.	Yes	Yes	Cohort	Yes	N/A	Calculated from date of LMP	Data from outpatient clinics	Yes	Yes	Yes
Kitchen et al	Yes	Yes	Cohort	No control group	N/A	Not documented	Data from outpatient clinics	Yes	No	No
Moisan et al.	Yes	Yes	Nested cohort	Yes	N/A	Reported by parents	Reported by parents	No	No	No
Peralta-Calcelen et al	Yes	Yes	Cross-sectional	Control group recruited in adolescence	N/A	Calculated from date of LMP and ultrasound measurements	Data from outpatient clinics	Yes	Yes	No
Persson et al.	Yes	Yes	Case control	N/A	Yes	Not documented	Data from outpatient clinics	No	No	Yes
Saigal et al	Yes	Yes	Cohort	Control group recruited at 8 years of age	N/A	Calculated from date of LMP and ultrasound measurements	Data from outpatient clinics	Yes	Yes	Yes
Sipola-Leppänen et al	Yes	Yes	Cohort	Yes	N/A	Calculated from date of LMP and ultrasound measurements	Data from outpatient clinics	Yes	Yes	Yes
Wehkalampi et al	Yes	Yes	Cohort	Yes	N/A	Not documented	Data from outpatient clinics	Yes	Yes	Yes

### Assessment of risk of bias at review level

All studies were published in peer reviewed journals. Regarding the possibility of publication bias, as most studies reported outcomes in addition to the onset of puberty, it is unlikely that there was a systematic bias against studies reporting either earlier or later onset of puberty after preterm delivery. Consultation with global experts in the field revealed only 1 study which had not been identified by our review, providing reassurance that we had successfully retrieved the majority of the available published evidence. However, consultation with these experts did reveal 4 datasets which contained information relevant to the aims of this review but had not been analyzed for our outcome of interest [43–46].

### Preterm birth and timing of puberty

The findings of the included studies are presented in Table 2. Studies varied in size, including between 12 [29] and 2748 [40] participants born preterm. Nine studies looked at cohorts of hospital born infants, [19, 30, 31, 33–38] and 7 recruited participants from childhood onwards. [28, 29, 32, 39–42] The timing of menarche was reported in all but one study, but there was otherwise wide variation in the summary measure used to describe the timing of puberty, making meaningful comparison of other measures challenging. The summary measure for the timing of menarche varied between studies, with 3 reporting median age, [19, 40, 42] 7 reporting mean age, [31, 33, 35, 36, 38, 39, 41] and the remaining 5 giving an alternative summary measure or not documenting one [28–30, 32, 34]. Of the 7 studies providing a mean, only 3 provided a standard deviation for both preterm and term groups.

**Table 2:** Full results from included studies

Country, Participant DOB (REF)	Study design	Participant selection	Gestation used to define prematurity (weeks)	Number of subjects		Form of summary measure	Age at menarche (years)		Age of onset of puberty for females (years)		Age of attainment of TS 2 for males (years)	
				Preterm	Term		Preterm	Term	Preterm	Term	Preterm	Term
Turkey, 1993–2003 (Atay et al)	Cross-sectional	Randomly selected healthy school girls.	Not specified	166	4702	Median	“Gestational age had no effect on the odds of being menarcheal”	12.74	“Gestational age had no effect on the odds of on attaining any of the pubertal stages”	TS 2–5 9.65, 10.10, 11.75, and 14.17 years, respectively		
India, 1968–1971 (Bhargava et al)	Cohort	Children born in urban hospital	37	79	176	Median	13.1	13.6	“Almost half the LBW were in TS 2 at 9.5 years”	28% at TS 2 at age 9.5	10.2	10.02
India, 1987–1989 (Chaudari et al)	Cohort	Infants discharged from Neonatal Unit	Not specified	147 (73 SGA, 74 AGA)	123 (33 SGA, 90 AGA)	Mean	12.5	12.7 (SGA) 12.8 (AGA)			SGA: 59% AGA: 73%	SGA: 61% AGA:64%
US and Puerto Rico, 1929–1975 (D’Aloisio et al.*.*)	Cohort	Women from the Sister Study with FH of Breast Cancer.	<36	767	17,365	Other	No significant difference in RR of menarche at different ages compared to controls					
France, 1925–1951 (Dossus et al.)	Cohort	Women recruited into E3N study	Not specified	2748	73,972	Median	0.07 years earlier	13				
Hawaii, 1986–1995 (Epplein et al.*.*)	Cross-sectional	Healthy females in adolescent maturation study.	<36	12	336	Other	Hazard ratio 1.61 (*p* value 0.16) with 36–41 weeks as reference.					
Australia, 1977–1982 (Ford et al.)	Nested cohort	VLBW (Mean gestation for BW < 1000 g =27 w; 1000–1499 g = 30w) and NBW controls	37	165	41	Other	At 14 yrs. of age: BW <1000 g: 15% BW: 1000–1500 g: 0%	6%	TS >3 at 14: BW <1000 g: 74% BW 1000–1500 g: 69%	75%	TS 3: BW < 1000 g: 88% BW 1000–1500 g:88%.	88%
USA, 1977–1979 (Hack et al)	Cohort	VLBW infants (mean 30 w) and controls	37	195	208	Other	12.4	12.3				
Hong Kong, 1997 (Hui et al)	Cohort	“Children of 1997” Birth Cohort	36	382	6984	Mean			9.88	9.68	11.71	11.67
Australia, 1966–1970 (Kitchen et al)	Cohort	Hospital born infants BW 500 g–1500 g	No controls	152	No controls	Mean	12.04	12.98 (standard Australian population)				
Canada, 1975–1976 (Moisan et al)	Nested cohort	Fifth grade females from public schools	Not specified	3022 overall	3022 overall	No data	“Prematurity had no association with early menarche”					
US, 1978–1984 (Peralta-Carcelen et al)	Cross-sectional	Adolescents born ELBW (mean 30 w) and term controls	37	53	53	Mean	11.15	11.45	TS 4/5: 88%	TS 4/5: 97%	“Testicular size not significantly different between groups”.	
Sweden, 1973–1977 (Persson et al)	Case control	Singleton births	37	139	688	Mean	13.2	13.1	Onset of pubertal growth spurt: 11.3	11.1	Onset of pubertal growth spurt: 12.0	12.1
Canada, 1977–1982 (Saigal et al)	Cohort	Infants born ELBW (and term controls	37	82	69	Mean	12.0	12.2				
Finland, 1986 (Sipola-Leppänen et al)	Cohort	Infants born early (GA <34 w) and late preterm (GA 34–36 w) and term controls	37	early preterm = 79 late preterm = 238	6325	Other			At age 16 54.3% early preterm had reached TS 4/5, 58.9% late preterm	At age 16 69.5% had reached TS 4/5	At age 16 65% early preterm had reached TS 4/5, 71.5% late preterm	At age 16 75.3% had reached TS 4/5
Finland, 1978–1985 (Wehkalampi et al)	Cohort	Infants born VLBW (SGA mean 32 w; AGA mean 28 w) and term controls	37	SGA = 35 AGA = 78	146	Mean	SGA:12.6 AGA:12.2	12.5			Voice break SGA:13.5 AGA:13.3	13.8

*Abbreviations*: *DOB* Date of Birth, *RR* Relative Risk, *w* weeks, *SGA* Small for Gestational Age, *AGA* Appropriate for Gestational Age, *BW* Birth Weight, *LBW* Low Birth Weight, *VLBW* Very Low Birth Weight, *ELBW* Extremely Low Birth Weight, *NBW* Normal Birth Weight, *TS* Tanner Stage

Only four out of 15 studies (Additional file 2: Table S1) presented data on age of menarche in participants at an age where the majority of them would be expected to have attained it (> 15 years). As menarche can be classified as a binary variable (ie attained or not), if studies assessed participants at the same age, we believe a comparison between the proportion of participants who had attained menarche in the preterm and term groups can be reasonably be interpreted as indicating a relative acceleration or deceleration in pubertal timing in the preterm (compared to term) group. In the more complicated situation where studies assessed median or mean age at menarche amongst the sub-group of females who had attained it by a particular age (e.g. 12 or 14 years), it is plausible that this summary measure could be skewed by an unequal distribution of this event in the two sub-groups, and in addition to the problem of missing information, could complicate a comparison between the results from different studies. This heterogeneity in the outcome measure, and the point at which it was measured, rule out a formal meta-analysis.

With regards the timing of menarche in females, 5 studies found that menarche occurred earlier in preterm girls, [19, 31, 35, 36, 38, 40] 8 found that there was no difference between the preterm and term groups, [28–30, 32–34, 41, 42], and 1 showed that menarche was later in those born preterm (+ 0.2 years), [39]. One study showed earlier menarche (−0.3 years) in the appropriate for gestational age (AGA) group, and later menarche in the small for gestational age (SGA) group, (+0.1 years) [39]. This data is summarized in Table 3. The five studies that showed earlier menarche in the preterm compared to term group found it to be a median of 0.3 years earlier (range- 0.94 to −0.07 years). The study with the largest effect [35] however did not have an internal control group and instead used a national average.

**Table 3:** Summary of results

Authors and year of study	Country	Sex of participants	Timing of menarche in females (years)	Onset of puberty in males (years)	Statistical summary measure (type)
Atay et al.	Turkey	Females only	No difference		No
Bhargava et al	India	Females and males	Earlier in preterm (0.5)	Later in preterm (0.18)	No
Chaudari et al	India	Females and males	Earlier in preterm (0.3)	Earlier in preterm (9.7% more attained)	No
D’Aloisio et al.	US	Females only	No difference		Yes (RR with 95% CI)
Dossus et al.	France	Females only	Earlier in preterm (0.07)		Yes (Beta value with 95% CI)
Epplein et al	US	Females only	No difference		Yes (HR with 95% CI)
Ford et al.	Australia	Females and males	No difference	No difference	No
Hack et al	US	Females only	No difference		Yes (T test)
Hui et al.	Hong Kong	Females and males	Onset of puberty later in preterm (0.2)	No difference	Yes (TR with 95% CI)
Kitchen et al	Australia	Females only	Earlier in preterm (0.94)		No
Moisan et al	Canada	Females only	No difference		No
Peralta-Carcelen	US	Females and males	Earlier in preterm (0.3)	No difference	No
Persson et al.	Sweden	Females and males	No difference	No difference	Yes (T test)
Saigal et al	Canada	Females only	No difference		Yes (mean with sd)
Sipola-Lapponen et al	Finland	Females and males	“Girls born preterm were at an earlier pubertal stage than controls”	No difference	Yes (χ2 test)
Wehkalampi et al.	Finland	Females and males	Earlier in AGA preterm (0.3) Later in SGA preterm (0.1)	Voice break earlier in AGA (0.5) and in SGA (0.3) preterm	Yes (T test)

*Abbreviations*: *RR* Relative risk, *HR* Hazard Ratio, *TR* Time Ratio, *CI* Confidence Interval, *sd* standard deviation

Seven studies examined the onset of puberty in girls, 6 using Tanner breast stages. Of these, 1 study found an earlier onset of puberty in preterm infants, [19].2 showed no difference, [34, 42] and 3 studies showed later onset of puberty [31, 37, 39]. One study used the timing of the onset of the pubertal growth spurt and found a later onset of puberty in the preterm group [41]. Eight studies examined the onset of puberty in boys, using different markers. Six studies used Tanner stages, [19, 31, 34, 37–39] 1 used the onset of the pubertal growth spurt, [41] and a further study used age at voice break [36]. Of these, 2 studies showed an earlier onset of puberty in boys born preterm, [36] 5 showed no difference, [31, 34, 37–39, 41] and 1 showed a later onset of puberty in those born preterm [19].

## Discussion

The published data available shows no clear association between being born premature and substantially earlier pubertal onset. There may be a subtle trend towards preterm females entering puberty earlier. Five out of the 16 studies showed earlier menarche after preterm birth, with a range of effect of between −0.07 to - 0.94 years. However, over half of the studies demonstrated no effect of gestational age on menarcheal timing. Other measures of female pubertal onset such as Tanner Stages showed no clear pattern. An inconsistent pattern was also seen in males, although it is hard to draw conclusions from the data as three different outcome measures were used to assess pubertal status.

### Factors affecting the risk of bias

#### Size of studies

There was wide variability in the size of studies. As we could not perform a meta-analysis, there is a risk that our findings could be skewed by unrepresentative smaller studies. However, the largest study identified [40] included 2748 participants born preterm and 73,972 term-born controls. This study showed a small, but statistically significant, difference between the two groups, with those born preterm achieving menarche a median of 0.07 years earlier, which is in keeping with the findings of the review as a whole. The next largest study [28] included 767 participants born preterm and 17,365 controls, and did not find any difference in the timing of menarche. The remainder of the studies included between 12 and 382 participants born preterm. Due to the heterogeneity of the data we could not perform a funnel plot, but tabulating the data shows there is no clear correlation between the size of the study and the direction or magnitude of the effect found (Table 4).

**Table 4:** Size of study and results

Authors and year of study	Number of preterm participants	Number of term participants	Timing of menarche (years)
Dossus et al.	2748	73,972	Earlier in preterm (0.07)
D’Aloisio et al.	767	17,365	No difference
Hui et al.	382	6984	Onset of puberty later in preterm (0.2)
Sipola-Leppänen et al.	317	6325	Onset of puberty later in preterm
Hack et al	195	208	No difference
Atay et al.	166	4702	No difference
Ford et al.	165	41	No difference
Kitchen et al	152	No controls	Earlier in preterm (0.94)
Chaudari et al	147	123	Earlier in preterm (0.3)
Persson et al.	139	688	No difference
Wehkalampi et al.	123	146	Earlier in AGA preterm (0.3) Later in SGA preterm (0.1)
Saigal et al	82	69	No difference
Bhargava et al	79	176	Earlier in preterm (0.5)
Peralta-Carcelen	53	53	Earlier in preterm (0.3)
Epplein et al	12	336	No difference
Moisan et al	Not specified	3022 overall	No difference

*Abbreviations*: *RR* Relative risk, *HR* Hazard Ratio, *TR* Time Ratio, *CI* Confidence Interval, *sd* standard deviation

#### Confounding factors

Both the risk of being born preterm and the risk of entering puberty at an earlier age may share a number of parental confounding factors. A number of studies adjusted for these, in particular parental socioeconomic status (5 studies), [19, 33, 38, 39, 41] education (5 studies), [19, 29, 36–38] and height (3 studies); [19, 36, 38] and maternal age (3 studies) [28, 39, 41]. It is possible that adjusting for these variables might attenuate any relationship found between preterm birth and risk of earlier menarche, and thus our data could be skewed by the studies which did not carry out any adjustment. However, examining the studies that adjusted for confounding factors showed that 38% of these (3/8) identified earlier puberty in those born preterm, compared to 29% (2/7) of those that did not, indicating that if confounding bias exists for these factors, there is no clear association in their relationship to preterm birth and earlier menarche.

#### Correcting for gestational age

Another potential source of bias is whether studies accounted for degree of prematurity, by correcting for gestational age at birth (number of weeks of prematurity subtracted from the chronological age). Only two studies performed this adjustment [36, 39]. One of these [36] found that those born preterm and at a birthweight appropriate for gestation entered puberty and attained menarche earlier, but that correcting for gestational age attenuated this effect. Conversely, the other study, [39] which showed that preterm birth was associated with later onset of puberty and menarche, found that correcting for gestational age removed this association. Together these studies show that correction for gestational age is unlikely to bias results significantly, as it had no clear effect in either direction.

#### Degree of prematurity and onset of puberty

Another factor that might affect the results was whether studies included those born extremely preterm, as they might be expected to go into puberty earlier if there is indeed a relationship between the intensity of adverse early life conditions and risk of earlier puberty. Four studies did not specify the gestational age at which they defined prematurity; [32, 38, 40, 42] in the remaining 12 studies, the gestational age of the participants ranged from 24 to 37 weeks. As a proxy for extreme prematurity, there were 6 studies which included participants with a very low birth weight (VLBW, <1500 g) [30, 34–36] or extremely low birth weight (ELBW, < 1000 g) [31, 33]. Of these, 2 studies (33%) showed that girls had earlier menarche, [31, 35, 36] a lower percentage than the 5/9 studies (56%) that did not include participants born with a VLBW/ELBW, thus suggesting no clear association between extreme prematurity and age at onset of puberty. It is likely that other causes of low birthweight also influence pubertal timing, rather than length of gestation alone. The fact that the study that categorized participants into AGA or SGA found differences in the timing of menarche [39] suggests that this might be a significant factor.

In addition to the factors discussed above, it is likely that other variables that we could not control for in our analysis, such as childhood growth, [47] probably have an equally, or perhaps more important role on pubertal timing [48]. Diet and body composition, which are inextricably linked to socioeconomic status, also play a significant role in the timing of an individual’s pubertal development. Several studies of both preterm and term-born cohorts have shown that obesity has a clear influence on pubertal timing, [36, 49] and according to the Developmental Origins of Health and Disease (DoHAD) hypothesis, both intrauterine and early life environments are important for later health outcomes [50]. There is insufficient information in the studies reviewed to examine the role of catch up growth and childhood weight gain on pubertal timing. Other important factors that cannot be overlooked include genetic and psychosocial factors, including exposure to stress and trauma (which have been linked to both earlier [51, 52] and later menarche), [53, 54] and the role of exposure to endocrine disruptors on later pubertal timing [55].

#### Limitations

There was marked heterogeneity in assessment of both the exposure and the outcome, and many studies did not calculate a statistical summary measure, limiting our ability to compare the studies, and meaning we could not perform a funnel plot. Similarly, as most studies did not present outcomes in the form of a mean with a standard deviation, we were unable to perform a meta-analysis. There was insufficient data within the articles to enable us to address all potential confounding factors. If this research question is to be investigated in further detail it would be beneficial to utilize the additional data contained within the large population- based datasets highlighted to us by experts in the field. These datasets are from the ALSPAC cohort in the United Kingdom [45], 2 cohorts of patients who formed part of trials in Australasia looking at the long term effects of antenatal corticosteroids, [43, 44], and a large birth cohort from Finland [46]. In order to perform a meta-analysis including this unpublished data, sourcing and standardization of the existing datasets would also be required to enable additional statistical analysis.

Another important factor in attempting to identify whether there is a stereotyped phenotypic response to a particular exposure is the homogeneity of the relevant population. In our case, although many preterm deliveries occur after the spontaneous onset of preterm labor, a substantial proportion are precipitated by infection, or are medically expedited for maternal or fetal indications [5]. Thus, it may be that classing all those born preterm into a single group and attempting to identify a response to the exposure of an early delivery is an over-simplification of what is likely to be a combination of complex biological mechanisms.

## Conclusion

The published evidence does not suggest that being born preterm in itself leads to a significant acceleration in the onset of puberty. This lack of evidence for a substantial effect should prove reassuring for public health purposes, and clinicians counseling parents of infants born preterm. To strengthen the evidence base to answer the question whether preterm birth is associated with the timing of puberty, further studies re-analyzing existing study data and including unpublished data from existing datasets will be required.

## Additional files

Additional file 1: **Appendix S1.** Review protocol. (DOCX 45 kb)

Additional file 2: **Table S1.** Age at participant review for included studies. (DOCX 12 kb)

## Abbreviations

AGA: Appropriate for gestational age
CASP: Critical appraisal study process
DoHAD: Developmental origins of Health and Disease
ELBW: Extremely low birth weight
SGA: Small for gestational age
VLBW: Very low birth weight
