# Supplementary material for: Preterm birth and the timing of puberty: a systematic review
Source: BMC Pediatr. 2018 Jan 8;18:3. doi: 10.1186/s12887-017-0976-8 (PMC5759269; doi:10.1186/s12887-017-0976-8)
Supplement: Supplementary file 1 — Appendix S1. Review protocol. (DOCX 45 kb) [file 12887_2017_976_MOESM1_ESM.docx]

**Additional file 1: Review Protocol**

**Premature Delivery and the Timing of Puberty**

1. **Background**

Survival of preterm infants born at early gestations is improving, and thus these patients are now surviving into adolescence and adulthood. However, the effect of premature delivery on the timing of puberty remains unclear. This has important implications for the long term health of this population. Earlier puberty is known to be linked to an increased risk of the metabolic syndrome and cardiovascular disease in adult life, as well to lower final height, and thus the potential to transmit these increased risks across generations. In addition, in females it is linked to an increased risk of breast cancer, and may be linked to an increased risk of depression and other psychopathology.

1. **Research question**

In adolescents (Population), what are the effects being born prematurely at <37 weeks (Exposure) versus being born at term (Comparison) on the timing of onset of puberty (Outcome), as reported in cohort, cross sectional or case control studies (Study design).

1. **Search Strategy**

This combined automated and manual search will use multiple search engines and databases (see Table a). Search terms are listed in the Appendix formatted for the various databases. We will follow through references meeting the inclusion criteria, use citation tracking to identify further relevant studies, and contact experts in the field for unpublished data and possible relevant publications.

**Table a:** Databases and Search engines

| **Database** |
| --- |
| Medline (via OVID interface) |
| Embase (via OVID interface) |
| Global Health (via OVID interface) |
| Global Health Library |
| Popline |

1. **Selection Criteria**
   1. Inclusion Criteria:
      1. Population: Pubertal and post-pubertal adolescents and adults
      2. Study setting: Outpatient settings
      3. Sampling method and study design: Cohort, cross sectional or case control studies. No language, publication date, or publication status restrictions will be imposed. Conference abstracts and journal papers will both be eligible.
      4. Sample size: No restriction
      5. Exposure: Premature delivery at <37 weeks, as defined as time from last menstrual period or gestation as calculated from antenatal ultrasound
      6. Results: Quantitative assessment (either by participants or study researchers) of commencement of puberty: in females the age at which menarche was recorded, and in males age at which they reached Tanner Stage 2.
   2. Exclusion Criteria:
      1. Study design: Case reports or opinion pieces.
      2. Exposure: Studies that reported on birthweight but not gestation for the patient population
      3. Results: Qualitative studies that did not provide data on onset of puberty
2. **Study selection**

Two researchers will independently carry out the search and assess whether studies meet in the inclusion and exclusion criteria. Disagreements between reviewers will be resolved by consensus. Figure 1 shows the process whereby studies will be selected.

1. **Assessment of Risk of Bias**

We will apply principles from the Critical Appraisal Skills Program (CASP) to assess the quality of each study. For each cohort study we will examine the following variables (table b) .

**Table b:** Sample Table for Quality Assessment

| Clearly focused study question? | Cohort recruitment acceptable?  (Cohort, cross sectional studies) | Case control appropriate study method? (Case control studies) | Exposure accurately measured? | Outcome accurately measured? | Confounding factors identified? | Confounding factors accounted for? | How precise is the estimate of risk? |
| --- | --- | --- | --- | --- | --- | --- | --- |
|  |  |  |  |  |  |  |  |

1. **Data Extraction**

One review author will extract data from the included studies, and the second author will check the extracted data. It will be inputted into Table c. Disagreements will be resolved by discussion between the two review authors, and if agreement cannot be reached, this decision will be made by a third author.

**Table c:** Sample Data Extraction Table

| Author,  Year | Country | Study design | Study Setting | Number of study subjects born preterm | Number of study subjects born at term | Mean age of menarche for subjects born preterm (female) | Mean age of attainment of Tanner Stage 2 for subjects born preterm (male) | Mean age of menarche for subjects born at term (female) | Mean age of attainment of Tanner Stage 2 for subjects born at term (male) |
| --- | --- | --- | --- | --- | --- | --- | --- | --- | --- |
|  |  |  |  |  |  |  |  |  |  |

1. **Synthesis**

The results from individual studies will be entered into a Forest plot for comparison, and if data is sufficiently homogenous a meta-analysis will be conducted to calculate a relative risk/odds ratio with a confidence interval.

1. **Study Limitations**

It is probable that there are few studies looking at this topic, and that our results may be skewed by a small number of trials with a low number of participants. The quality of the trials may be variable, and the data may be so heterogenous that a substantial meta-analysis is not possible.

**Figure 1:** Sample Flowchart for Data Extraction

Records after duplicates removed
(n = )

Additional records identified through other sources
(n = )

## Identification

## Screening

Records identified through database searching
(n = )

Records excluded

Records screened
(n = )

Records excluded- did not describe:

## Eligibility

Full-text articles assessed for eligibility
(n = )

Studies included in qualitative synthesis
(n = )

## Included

Studies included in quantitative synthesis
(n = )

Appendix: Search Terms

**Pubmed/Medline**

Premature birth/ or infant, low birth weight/ or infant, premature/ or preterm.mp or intrauterine.mp

AND

Pubert*.mp OR menarche.mp

AND

Follow up studies/ or age.mp or onset.mp

🡪 Limit to humans

**Embase**

Prematurity/ OR low birth weight/ OR preterm.mp OR intrauterine.mp

AND

Pubert*.mp OR menarche.mp

AND

Follow up/ or age.mp or onset.mp

🡪 Limit to humans

Crib for Medline/Embase search in OVID:

(Premature birth/ or infant, low birth weight/ or infant, premature/ or prematurity/ or low birth weight/ or preterm.mp or intrauterine.mp) AND (Pubert* OR menarche) AND (Follow up studies/ or follow up/ or age or onset)

**Global Health**

Premature birth OR infant, low birth weight OR infant, premature OR prematurity OR low birth weight OR preterm OR prematur* OR low birth weight

AND

Pubert* OR menarche

AND

Follow up studies OR follow up OR age OR onset

Crib for OVID:

(Premature birth OR infant, low birth weight OR infant, premature OR prematurity OR low birth weight OR preterm OR prematur* OR low birth weight) AND **(**pubert* OR menarche) AND **(**Follow up studies OR follow up OR age OR onset)

**Global Health Library**

preterm OR prematur* OR low birth weight

AND

pubert* OR menarche

AND

age OR onset

**Popline**

preterm OR prematur* OR low birth weight

AND

pubert* OR menarche

AND

age OR onset
