# Supplementary material for: Preterm birth and the timing of puberty: a systematic review
Source: BMC Pediatr. 2018 Jan 8;18:3. doi: 10.1186/s12887-017-0976-8 (PMC5759269; doi:10.1186/s12887-017-0976-8)
Supplement: Supplementary file 2 — Table S1. Age at participant review for included studies. (DOCX 12 kb) [file 12887_2017_976_MOESM2_ESM.docx]

Additional file 2: Table S1: Age at participant review for included studies

| Country, Participant year of birth (ref) | | Age at participant review (years) |
| --- | --- | --- |
|  | |  |
| Turkey, 1993-2003 (Atay *et al*) | | <15 but stratified by age |
| India, 1968-1971 (Bhargava *et al*) | | Measured at 14yrs |
| India, 1987 – 1989 (Chaudari *et al*) | | Followed till 12yrs |
| US and Puerto Rico, 1929-1975 (D’Aloisio *et al*) | | 35-59 yrs |
| France, 1925-1951 (Dossus *et al*) | | 40-65 yrs |
| Hawaii, 1986- 1995 (Epplein *et al*) | | Median 12.8 yrs (continued to see till menarche) |
| Australia, 1977-1982 (Ford *et al*) | | Up to 14 yrs |
| USA, 1977-1979 (Hack *et al*) | | 20 yrs |
| Hong Kong, 1997 (Hui *et al*) | | 11 yrs |
| Australia, 1966-1970 (Kitchen *et al*) | | Mean 14.5 yrs |
| Canada, 1975-1976 (Moisan *et al*) | | 10-11 yrs |
| US, 1978 – 1984 (Peralta-Carcelen *et al*) | | Mean 14.85 yrs |
| Sweden, 1973-1977 (Persson *et al*) | | 16 yrs |
| Canada, 1977-1982 (Saigal *et al*) | | 12-16 yrs |
| Finland, 1978-1985 (Wehkalampi *et al*) | | Mean age for last measurement 16.4 yrs |
|  |  |  |
